# Supplementary data for a model-based health economic evaluation on lung cancer screening with low-dose computed tomography in a high-risk population

**DOI:** 10.1016/j.dib.2020.105999

**Published:** 2020-07-04

**Authors:** Yihui Du, Grigory Sidorenkov, Marjolein A. Heuvelmans, Harry J.M. Groen, Karin M. Vermeulen, Marcel J.W. Greuter, Geertruida H. de Bock

**Affiliations:** a University of Groningen, University Medical Center Groningen, Department of Epidemiology, Groningen, The Netherlands; b University of Groningen, University Medical Center Groningen, Department of Pulmonary Diseases, Groningen, The Netherlands; c University of Groningen, University Medical Center Groningen, Department of Radiology, Groningen, The Netherlands

**Keywords:** Low-dose computed tomography, Lung neoplasm, Mass screening, Micro-simulation model, Economic evaluation

## Abstract

This supplementary data is supportive to the research article entitled ‘Cost-effectiveness of lung cancer screening with low-dose computed tomography (LDCT) in heavy smokers: A micro-simulation modelling study’ (Yihui Du et al. 2020). This supplementary contains a description of the model input and the related model output data that were not included in the research article. The input data used for the tumour growth model and the self-detected tumour size model are provided. The output data of this article include the data used for cost-effectiveness analysis of lung cancer LDCT screening with the Dutch and international discount rates, the data of the sensitivity analysis, and the data of the model validation.

Specifications table

**Table:** 

Subject	Public health and health policy
Specific subject area	Health economic evaluation of lung cancer screening
Type of data	Microsoft office excel worksheet tabs
How data were acquired	Collected from published literature. Model output.
Data format	Raw
Parameters for data collection	The characteristics of the target population.
Description of data collection	The input data to the model was independently collected from online publications. The data for the health economic evaluation were the output data of the model.
Data source location	Institution: University of Groningen City: Groningen Country: the Netherlands
Data accessibility	Repository name: Mendeley Data Direct URL to data: http://dx.doi.org/10.17632/kg5yjcs7b4.1
Related research article	Yihui Du, Grigory Sidorenkov, Marjolein A. Heuvelmans, Harry J. M. Groen, Karin M. Vermeulen, Marcel J.W. Greuter, Geertruida H. de Bock, Cost-effectiveness of lung cancer screening with low-dose computed tomography in heavy smokers: A micro-simulation modelling study, European Journal of Cancer. 2020; 135: 121–129.

## Value of the data

• The data provide the details of model input regarding the cost-effectiveness of lung cancer screening, which can facilitate the readers' understanding of the model structure.
• The data provide the details of model output of validation and various lung cancer screening scenarios, which are helpful to understand the related research article.
• All researchers and readers who focus on model-based cost-effectiveness of lung cancer screening can benefit from these data.
• The data could enrich the readers' knowledge about the model-based health economic evaluation of lung cancer screening.

## 1 Data

The provided data are supplementary to the associated research article [1]. Data provided in this article include the model input data that were not included in the research article and the model output data for the health economic evaluation on lung cancer screening with low-dose computed tomography (LDCT). The accompanying data file ‘Cost-effectiveness_Lung cancer screening_Dataset’ is provided and available in the data repository [2].

### 1.1 Model input data

The input data used for the tumour growth and self-detection model are provided in the data repository. The distribution of volume doubling times (VDT) of lung cancers was extracted from the publication of Henschke et al. [3]. The extracted data and the derived input values used in the model are presented in the worksheet tab “tumour growth” of the excel file “Input_data.xlsx”. The distribution of self-detected tumour sizes was extracted from the publication of Rami-Porta et al. [4]. The extracted data and the derived input values used in the model are presented in the worksheet tab “Self-detected tumour size” of the excel file “Input_data.xlsx”.

### 1.2 Model output data

The following analyses were applied in the original research article: the cost-effectiveness analysis of lung cancer screening with a Dutch discount rate, the cost-effectiveness analysis of lung cancer screening with an international discount rate, the sensitivity analysis and the model validation. The datasets related to these four analyses are provided. The output data of the model are presented separately for men and women. Each excel file contains the output data of 10 iterations of the model and the average was calculated. The datasets are described in order.

#### 1.2.1 Cost-effectiveness with the Dutch discount rate

The data used for the cost-effectiveness analysis of lung cancer screening with the Dutch discount rate are provided. The following tables of the original research article are based on these data, including “Table 2″, “Table 3″ and “Table S8” [1]. The data of 18 scenarios for men and women are presented in the excel file “Output_Main results_Men.xlsx” and “Output_Main results_Women.xlsx”, respectively. The name of each worksheet tab indicates the performed scenario. For example, “A-50–75″ signifies annual screening from 50 to 75 years old, and “B-50–75″ signifies biennial screening from 50 to 75 years old. In each scenario, the following data are included for screening and no screening: the size of the simulated population, the number of lung cancers in the screened population, the number of participants with a screen-detected lung cancer, the number of participants that died from lung cancer, the number of life years, the number of interval lung cancers, the number of false positive results and the total costs. In addition, a summary table of the output of each scenario is provided, which includes the mortality reduction compared to no screening, the average cost-effectiveness ratio (ACER) per averted lung cancer death, the ACER per life years gained (LYG) and the number of radiation-induced lung cancers. The cost and the discounted LYG are the result after discounting by 4% for cost and 1.5% for LYG.

#### 1.2.2 Cost-effectiveness with the international discount rate

The data used for the cost-effectiveness analysis of lung cancer screening with the international discount rate are provided. A discount rate of 3% for both cost and LYG was applied. The “Table S9” of the original research article is based on these data [1]. The data of 18 scenarios for men and women are presented in the excel file “Output_Main results_Men_International discount.xlsx”, “Output_Main results_Women_International discount.xlsx”, respectively. For the description of the content of the two excel files we refer to “1.2.1 Cost-effectiveness with the Dutch discount rate”.

#### 1.2.3 Sensitivity analysis

The data used for the sensitivity analysis of the original research article are provided. The scenarios for the sensitivity analysis were described in the original research article [1]. The “Table S9”, “Figure S2” and “Figure S3” of the original research article are based on these data [1]. The data for men and women are presented in the excel files “Output_Sensitivity analysis_Men_A-55–80.xlsx” and “Output_Sensitivity analysis_Women_B-50–80.xlsx”, respectively. The name of each worksheet tab indicates the variable that was varied in the sensitivity analysis. For the content of the worksheet tabs we refer to “1.2.1 Cost-effectiveness with the Dutch discount rate”.

#### 1.2.4 Model validation

The data used for the model validation of the research article are provided in the excel file “Output_Validation”. The data of the number of screen-detected lung cancers, the number of interval lung cancers and the size distribution of the screen-detected tumours in the first and second screening rounds are presented in 3 worksheet tabs with the corresponding names. The excel file contains the data for men and women separately, as well as the combined data. The “Table S5” and “Table S6” of the original research article are based on these data [1].

## 2 Experimental design, materials and methods

The related research article was designed to evaluate the cost-effectiveness of lung cancer screening with LDCT in a high-risk population using a micro-simulation model [1]. The design, materials and methods are clearly described in the research article. Briefly, the micro-simulation model SiMRiSc was used. This model has previously successfully been used to evaluate the cost-effectiveness of breast cancer screening programs. It was adapted for the purpose of lung cancer LDCT screening. The model was validated by comparing the simulated outcomes to the observed data from the Dutch-Belgian Randomized Lung Cancer Screening (NELSON) trial. The evaluated screening scenarios combined different key characteristics of LDCT screening strategies: screening interval, and start and stop age of screening. The evaluated outcomes included the average cost-effectiveness ratio, incremental cost-effectiveness ratio, lung cancer mortality reduction, life years gained, number of lung cancer deaths averted, interval lung cancers, false positives, radiation-induced lung cancers and additional costs relative to no screening. One-way sensitivity analyses were performed to explore the parameters uncertainty of the most cost-effective scenarios. The technical details of the model were described in the supplementary of the original research article [1].

## Declaration of Competing Interest

The authors declare that they have no known competing financial interests or personal relationships which have, or could be perceived to have, influenced the work reported in this article.
